# Microbe‐responsive human γδ T cells: the peculiar case of *Staphylococcus aureus*

**DOI:** 10.1111/imcb.70060

**Published:** 2025-09-22

**Authors:** Matthias Eberl, Manuel Mata Forsberg, James E. McLaren, Eva Sverremark‐Ekström

**Affiliations:** ^1^ Division of Infection and Immunity, School of Medicine Cardiff University Cardiff UK; ^2^ Systems Immunity Research Institute Cardiff University Cardiff UK; ^3^ Department of Molecular Biosciences The Wenner‐Gren Institute, Stockholm University Stockholm Sweden

**Keywords:** bacteria, γδ T cells, immunity, infection, *Staphylococcus aureus*, superantigens

## Abstract

Vγ9/Vδ2 T cells represent the largest γδ T‐cell population in human blood and possess a unique responsiveness towards microbial organisms by sensing the metabolite (*E*)‐4‐hydroxy‐3‐methyl‐but‐2‐enyl pyrophosphate (HMB‐PP) in the context of the butyrophilin family members BTN2A1 and BTN3A1. Curiously, the bacterium *Staphylococcus aureus* does not produce HMB‐PP but appears to be capable of inducing activation, cytokine expression and proliferation of Vγ9/Vδ2 T cells regardless, through a largely unknown mechanism. We here provide a comprehensive review of the existing literature around Vγ9/Vδ2 T‐cell responses to *S. aureus* and discuss potential pathways, ligands and biological functions.

## Graphical abstract

Human Vγ9/Vδ2 T cells display a unique responsiveness towards microbial organisms producing the metabolite HMB‐PP. However, *Staphylococcus aureus* does not possess HMB‐PP but can induce activation, cytokine expression and proliferation of Vγ9/Vδ2 T cells regardless. We here discuss potential pathways, ligands and biological functions in this response to *S. aureus*.

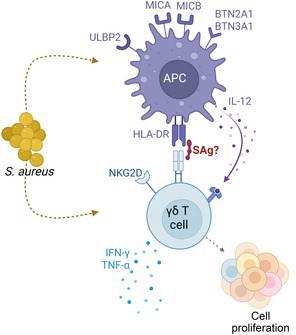

Four decades after their discovery, human γδ T cells continue to puzzle immunologists.^1^ Defying attempts to shoebox them into the canonical MHC‐restricted system of antigenic peptide‐specific conventional CD4^+^ and CD8^+^ T cells, Vγ9/Vδ2^+^ γδ T cells soon emerged as an “unconventional” and promiscuous lymphocyte subset capable of responding to a wide range of pathogens. Vγ9/Vδ2 T cells typically comprise only 0.5%–5% of human peripheral blood T cells but can expand rapidly upon microbial stimulation *in vitro* and during acute infections.^2^ The responsible antigenic compounds turned out to be phosphorylated nonpeptidic molecules (often referred to as “phosphoantigens”): the isoprenoid building block isopentenyl pyrophosphate (IPP) that is produced by all living cells, and the 10,000 times more potent direct precursor of IPP, (*E*)‐4‐hydroxy‐3‐methyl‐but‐2‐enyl pyrophosphate (HMB‐PP) that is restricted to microbes possessing the 2‐*C*‐methyl‐d‐erythritol 4‐phosphate (MEP) pathway of isoprenoid biosynthesis.^3^, ^4^ The molecular mechanism underlying the recognition of HMB‐PP is only beginning to be understood – rather than being presented on the cell surface like a classical T‐cell antigen, HMB‐PP acts intracellularly by “glueing” together the intracellular domains of BTN2A1 and BTN3A1 and triggering a conformational change of these butyrophilin family members on the cell surface that is then sensed by the Vγ9/Vδ2 T‐cell receptor.^5^ Whether microbially produced IPP plays a similar role under physiological conditions remains unclear.

## IDENTIFICATION OF MICROBIAL HMB‐PP AS A NATURAL ACTIVATOR OF HUMAN γδ T CELLS

The first link between the MEP pathway and Vγ9/Vδ2 T cells was made in 1999 when Jomaa *et al*. reported that only bacteria possessing the alternative MEP pathway of isoprenoid biosynthesis, but not bacteria utilizing the classical mevalonate pathway, were capable of inducing the proliferation of Vγ9/Vδ2 T cells in human peripheral blood mononuclear cell (PBMC) cultures, regardless of the amount of IPP present in bacterial preparations.^6^ Belmant *et al*. described the responsible compound as 3‐formyl‐1‐butyl pyrophosphate, isolated from mycobacteria^7^; however, the structural identity and bioactivity of 3‐formyl‐1‐butyl pyrophosphate could not be confirmed.^8^ Instead, Hintz *et al*. and Reichenberg *et al*. purified the natural Vγ9/Vδ2 T‐cell activator from *Escherichia coli* and identified it as HMB‐PP, a novel bacterial metabolite and previously unknown intermediate of the MEP pathway,^9^ and validated the bioactivity of HMB‐PP by chemical synthesis.^10^

Davey *et al*. demonstrated that only HMB‐PP producing, but not HMB‐PP deficient, bacteria stimulated Vγ9/Vδ2 T cells upon phagocytosis by neutrophils, presumably by releasing HMB‐PP into the culture medium from where it is then taken up by monocytes and “presented” to Vγ9/Vδ2 T cells.^11^ Comprehensive proof for the key role of HMB‐PP in triggering Vγ9/Vδ2 T‐cell responses came from experiments using genetically engineered bacteria where overproduction of HMB‐PP in *E. coli*, *Listeria monocytogenes*, *Listeria innocua*, *Mycobacterium smegmatis* and *Salmonella enterica* increased the organisms' potential to activate Vγ9/Vδ2 T cells, compared with the parental strains (reviewed in^12^). Genetic deletion of MEP pathway enzymes upstream of the production of HMB‐PP in *E. coli* and *Listeria monocytogenes* abrogated the microbial bioactivity on Vγ9/Vδ2 T cells, as did inhibition of the MEP pathway using the antibiotic fosmidomycin in both bacteria and malaria parasites (reviewed in^12^). Finally, Liuzzi *et al*. provided *in vivo* evidence for a correlation between the presence of the MEP pathway and Vγ9/Vδ2 T‐cell responses by showing that Vγ9/Vδ2 T cells preferentially accumulate at the site of infection in patients with acute peritonitis caused by bacteria producing HMB‐PP, but not by HMB‐PP‐deficient bacteria.^13^ Case closed? If only it were that simple.

## HUMAN γδ T‐CELL RESPONSES TO *STAPHYLOCOCCUS AUREUS*

The original lists of bacterial species that did not trigger a detectable response by Vγ9/Vδ2 T cells *in vitro* included *Staphylococcus aureus*, in agreement with its lack of the MEP pathway and hence its inability to produce HMB‐PP. This was true both in human PBMC cultures exposed to low molecular weight extracts of *S. aureus*,^6^ and in co‐cultures of purified Vγ9^+^ T cells and neutrophils harboring live *S. aureus*.^11^ However, contradicting findings began to emerge suggesting that under certain conditions Vγ9/Vδ2 T cells can indeed respond to *S. aureus* (Table 1).

**Table 1:** Summary of studies reporting human γδ T‐cell responses to *Staphylococcus aureus*.

Stimulus	Experimental set‐up	γδ T‐cell response	Read‐out	Ref.
Whole bacteria				
Live bacteria	γδ T‐cell clones + monocytes/monocyte‐derived DCs	Yes	IFN‐γ, TNF‐α	[20]
Live bacteria	Purified Vγ9^+^ T cells + monocytes + neutrophils	No	Proliferation, CD69, TNF‐α	[11]
Live bacteria	Purified γδ T cells + monocyte‐derived DCs	Yes	CD69, IFN‐γ	[17]
Heat‐killed bacteria	PBMC	Yes	CD25, CD69, IFN‐γ (TNF‐α)	[15]
Acute infection	Neonatal sepsis patients	Yes	TRD repertoires	[18]
Bacterial extracts				
LMW	PBMC	No	Proliferation	[6]
LMW	Peritoneal leukocytes	Yes	CD69, TNF‐α	[13]
CSF	PBMC	Yes	IFN‐γ, CD107a	[16]
Superantigens				
SEA	PBMC	Yes	IFN‐γ	[16]
SEA	γδ T‐cell clones + EBV‐transformed B cells	Yes	Proliferation	[34]
SEA, SEB, TSST‐1	γδ T‐cell clones + EBV‐transformed B cells	Yes	Proliferation	[32]
SEA, SEB, SED, TSST‐1	Purified γδ T cells + EBV‐transformed B cells	Yes	Proliferation	[33]

CFS, cell‐free supernatant; DCs, dendritic cells; EBV, Epstein–Barr virus; LMW, low molecular weight extract; TRD, T‐cell receptor δ locus.

Liuzzi *et al*. studied the activation of Vγ9/Vδ2 T cells among peritoneal leukocytes from individuals undergoing peritoneal dialysis as life‐saving renal replacement therapy and reported a positive response to low molecular weight extracts from a range of HMB‐PP producing Gram‐positive and Gram‐negative bacteria, as expected, but also from *S. aureus*. Other HMB‐PP‐deficient species such as *Enterococcus faecalis* and *Streptococcus pneumoniae* were inactive in those experiments.^13^ While the Vγ9/Vδ2 T‐cell response to HMB‐PP producing bacteria could be abrogated using the anti‐BTN3A blocking antibody clone 103.2, in line with the now established recognition of HMB‐PP in the context of BTN3A1 and BTN2A1,^14^ unfortunately no such blocking experiments were carried out with regard to *S. aureus*.^13^

Corroborating the view that Vγ9/Vδ2 T cells can be activated in an HMB‐PP‐independent manner, Suen *et al*. showed positive responses by Vγ9/Vδ2 T cells in human PBMCs cultured in the presence of heat‐killed *E. coli*, *Mycobacterium tuberculosis* and *S. aureus*, as well as the HMB‐PP‐deficient yeast *Candida albicans*.^15^ Johansson *et al*. demonstrated that exposure of PBMCs to bacterial supernatants from *S. aureus*, but not to HMB‐PP‐deficient *Lactobacillus rhamnosus* or *Lactobacillus reuteri*, stimulated IFN‐γ production by γδ T cells.^16^ This response could be reduced by anti‐IL‐12 blocking antibodies, suggesting an indirect effect via secretion of IL‐12 by accessory cells such as monocytes present in the PBMC cultures. Cooper *et al*. extended these findings by co‐culturing human γδ T cells with monocyte‐derived dendritic cells (DCs) that had been infected with live bacteria, demonstrating that Vδ2^+^ T cells, but not those with Vδ1^+^ or Vδ3^+^ T‐cell receptors (TCRs), readily responded to various *S. aureus* strains under these conditions.^17^ These responses depended on cell–cell contact between γδ T cells and infected DCs and could be partially inhibited using either anti‐TCRγδ or anti‐IL‐12 blocking antibodies, suggesting a combination of direct and indirect effects.^17^

Finally, physiological evidence that Vγ9/Vδ2 T‐cell responses to *S. aureus* occur in natural infections was recently provided by Giannoni *et al*., showing that in children younger than two years, foetal‐derived Vγ9/Vδ2 TCR clonotypes expand in a pathogen‐specific manner during blood culture‐proven sepsis caused by both *E. coli* and *S. aureus*, but not by *Streptococcus pneumoniae*, suggesting a direct recognition of *S. aureus* through the Vγ9/Vδ2 TCR.^18^ Clowry *et al*. observed an expansion of systemic γδ T cells in children with atopic dermatitis and *S. aureus* skin infection and speculated that this may represent a compensatory response in the setting of conventional αβ T‐cell suppression.^19^

## HUMAN γδ T‐CELL RESPONSES TO CELLULAR STRESS LIGANDS

The molecular and cellular mechanisms of how Vγ9/Vδ2 T cells respond to *S. aureus* remain to be unveiled. Kistowka *et al*. suggested that dysregulation of the host mevalonate pathway in antigen‐presenting cells infected with *S. aureus* or *E. coli* may result in Vγ9/Vδ2 T‐cell responses to bacteria.^20^ However, this would not explain why others have failed to induce Vγ9/Vδ2 T‐cell responses to other HMB‐PP‐deficient live bacteria such as streptococci or the Gram‐negative bacterium *Chryseobacterium indologenes*.^11^, ^17^

**Figure 1:**
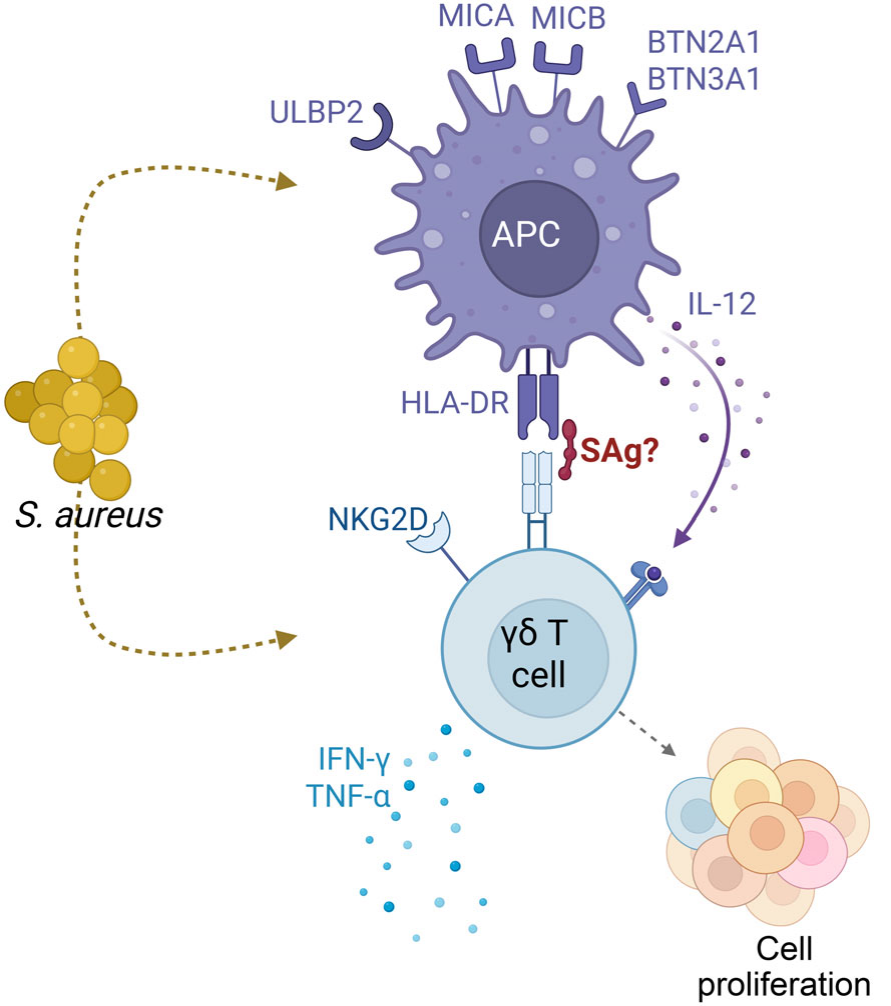
Potential direct or indirect activation of human γδ T cells by *Staphylococcus aureus*.

Although Vγ9/Vδ2 T cells respond mainly towards HMB‐PP, additional modes of activation exist.^1^ Cellular stress can induce expression of endogenous self‐ligands, several of which may act on γδ T cells in a TCR‐dependent manner.^21^ Under certain conditions, Vγ9/Vδ2 T cells have been reported to recognize ectopically expressed self‐proteins such as the heat shock protein HSP60, F1‐ATPase (a portion of the cellular ATP synthase) and apolipoprotein A‐I, as well as several NKG2D ligands.^22^, ^23^, ^24^, ^25^, ^26^, ^27^, ^28^ Finally, it cannot be excluded that *S. aureus* may be able to produce as yet unknown metabolites other than HMB‐PP, IPP or related isoprenoid precursors, with similarly potent activity on Vγ9/Vδ2 T cells (Craig T. Morita, personal communication). Together, these intriguing observations offer plausible alternative mechanisms of how Vγ9/Vδ2 T cells might respond towards HMB‐PP‐negative bacteria such as *S. aureus* and highlight the complexity of how antimicrobial immune responses are induced and regulated (Figure 1).

## HUMAN γδ T‐CELL RESPONSES TO STAPHYLOCOCCAL SUPERANTIGENS

Many pathogenic bacteria possess highly sophisticated strategies for evading innate and adaptive immune cells. To this extent, *S. aureus* has established an arsenal of virulence factors that counteract or even kill T cells, including pore‐forming toxins and highly potent enterotoxins called superantigens. *S. aureus* encodes at least 26 distinct superantigens that activate αβ T cells by crosslinking the αβ TCR with MHC class II molecules on antigen‐presenting cells (APCs), resulting in upregulation of activation markers, excessive cytokine release and polyclonal T‐cell proliferation.^29^, ^30^ Each strain of *S. aureus* typically carries multiple superantigen genes, which are often located on mobile genetic elements, leading to considerable variability and striking differences in immunogenicity between isolates and adding to the complexity of characterizing the interaction between *S. aureus* and the human immune system.^30^

Although staphylococcal enterotoxins (SEs) and toxic shock syndrome toxin‐1 (TSST‐1) have been studied with regard to their potential to activate γδ T cells, a general consensus on whether this occurs in a manner similar to that of conventional αβ T cells has yet to be reached. In 1990, Rust *et al*. described the cytotoxicity of Vγ9^+^ T‐cell clones against SEA‐coated target cells,^31^ while Spertini *et al*. showed that Vδ1^+^ and Vδ3^+^ T‐cell clones proliferated in response to SEA, SEB or TSST‐1‐stimulated accessory cells.^32^ This effect was dependent on the presence of MHC class II, as verified by Ramesh *et al*.^33^ Later on, Morita *et al*. identified the presence of an N‐terminal region within SEA that could potentially bind the Vγ9 chain within Vγ9/Vδ2 TCRs, although this was not confirmed structurally.^34^ Recently, Uzunçayir *et al*. demonstrated a somewhat weak but significant binding affinity between SEA and a chimeric γδ/αβ TCR receptor, suggesting that γδ T cells may indeed be activated by SEs through crosslinking of TCR‐MHC class II, similar to that of conventional αβ T cells (Figure 1).^35^

However, this notion that superantigen‐mediated effects on γδ T cells are dependent on TCR‐MHC class II crosslinking remains debatable, as SEA‐stimulated monocytes are unable to induce IFN‐γ production in γδ T cells in the absence of αβ T cells and cell‐to‐cell contact, according to Mata Forsberg *et al*.^36^ In the same study, superantigen‐mediated γδ T‐cell activation was shown to be IL‐12 dependent, following on from the original observation by Johansson *et al*.,^16^ and was consistent across γδ T cells bearing different Vδ chain pairs (Vδ1, 2 or 3) while their activation kinetics were markedly delayed compared with αβ T cells.^36^ Furthermore, SEA‐exposed macrophages failed to induce proliferation in γδ T cells even after 5 days of co‐culture, while causing a profound proliferative burst among αβ T cells (M.M.F. & E.S.E., unpublished data). Collectively, this implies that SE‐mediated effects on γδ T‐cell‐derived IFN‐γ production and proliferation are likely to be indirect and secondary to the direct αβ T‐cell response.

These discrepancies regarding how γδ T cells are activated by superantigens are also likely to be a result of several indirect factors. Firstly, Fikri *et al*. demonstrated that bovine WC1^+^ γδ T cells proliferate more in response to superantigen stimulation in the presence of APCs expressing high levels of the costimulatory molecules CD80 and CD86,^37^ the latter of which is capable of directly binding SEB.^38^ Indeed, antibody‐based simultaneous blocking of CD80 and CD86 resulted in a complete loss of γδ T‐cell proliferation, further indicating a requirement for co‐stimulation in the induction of SE‐mediated γδ T‐cell responses.

Secondly, older studies commonly used Epstein–Barr virus (EBV) transformed B cells or irradiated APCs in proliferation assays dependent on radioactive thymidine incorporation. In those cases, it is difficult to rule out the possibility that γδ T cells may sense and respond to altered self or stress‐induced ligands, rather than being directly activated by the toxin. Radiation induces various forms of cellular stress, for example DNA damage and metabolic alterations, which have the potential to result in the surface expression of stress‐induced self‐ligands. These could then be sensed by the immune system, in particular by γδ T cells.^21^, ^39^ Together with SE‐mediated γδ TCR engagement, these irradiation‐induced stress ligands could very well result in a dual signal environment sufficient to trigger T‐cell activation and subsequent proliferation. γδ TCR‐mediated cytolytic activity was, in fact, shown by Bessoles *et al*. to be enhanced in the presence of NKG2D ligands expressed on target cells.^22^ Similarly, Morita *et al*. observed that SEA‐induced proliferation of the γδ T‐cell line JN.23 occurred only when APCs were fixed with glutaraldehyde, attributing this effect to the generation of polymeric aldehydes on the APC surface.^34^

Thirdly, a dependency on IL‐2 was made evident by Fikri *et al*.^37^ Studies of γδ T‐cell responses to SEs commonly used clonal γδ TCR cell lines, often Jurkat cell lines, with a capacity to secrete IL‐2 upon stimulation. Maintaining Jurkat cell lines *in vitro* requires continuous re‐stimulation, which may even result in the constitutive expression of IL‐2.^40^ Interestingly, IL‐2 production was only observed within αβ T cells and not in the γδ T cells upon SE stimulation of human PBMC,^36^ further suggesting an indispensable role for αβ T cells in SE‐mediated activation of γδ T cells.

Finally, γδ T cells are present at relatively high frequencies at a young age and are believed to be functionally mature and therefore important contributors to infant immunity. Nevertheless, γδ T cells from young children respond poorly towards SEs, as do conventional αβ T cells. In fact, we only observed γδ T‐cell‐derived IFN‐γ production when conventional αβ T cells responded as well.^36^ This age‐dependent responsiveness towards SEs was found to be linked to the induction of monocyte‐derived IL‐12, which similarly increases with age.^36^

## OUTLOOK

The jury is still out as to how, and whether, human γδ T cells respond directly or indirectly to *S. aureus*. Given this uncertainty and inconsistency in the literature, we encourage researchers in the field to investigate this phenomenon closer, and in all their experiments to provide the exact details regarding origin, strain specificity and superantigen expression of the *S. aureus* cultures used, especially when working with clinical isolates. The involvement of the γδ TCR in *S. aureus* responses has not formally been demonstrated other than in some anti‐TCRγδ blocking experiments conducted by Cooper *et al*.^17^ A better definition of the host co‐factors involved in human γδ T‐cell responses to *S. aureus*, including the role of BTN2A1/BTN3A1 and related molecules, is urgently needed, as is the characterization of the bacterial genes and pathways influencing the bioactivity. Ultimately, the outcome of such responses remains to be clarified, in the light of increasing evidence for a protective role of mouse γδ T cells in *S. aureus* infection models.^41^, ^42^, ^43^ Do human γδ T cells contribute to the control and clearance of *S. aureus* infections? Or is the triggering of human γδ T cells, especially when involving superantigens, an effective means utilized by *S. aureus* to undermine and evade the host immune response? It will be interesting to see how the answers to these questions will help guide the development of better treatments and vaccines against *S. aureus*, an organism which remains a significant cause of morbidity and mortality in humans, responsible for both mild and severe manifestations including skin, soft tissue and bloodstream infections, pneumonia and endocarditis.

## AUTHOR CONTRIBUTIONS

**Matthias Eberl:** Conceptualization; writing – original draft; writing – review and editing. **Manuel Mata Forsberg:** Writing – review and editing; writing – original draft; visualization. **James E. McLaren:** Writing – original draft; writing – review and editing. **Eva Sverremark‐Ekström:** Writing – original draft; writing – review and editing.

## CONFLICT OF INTEREST

The authors declare no conflicts of interest.

## Data Availability

Data sharing not applicable to this article as no datasets were generated or analysed during the current study.
